# Supplementary material for: Minibands in twisted bilayer graphene probed by magnetic focusing
Source: Sci Adv. 2020 Apr 17;6(16):eaay7838. doi: 10.1126/sciadv.aay7838 (PMC7164947; doi:10.1126/sciadv.aay7838)
Supplement: aay7838_SM.pdf [file aay7838_SM.pdf]

Science Advances
AAAS

advances.sciencemag.org/cgi/content/full/6/16/eaay7838/DC1

Supplementary Materials for

Minibands in twisted bilayer graphene probed by magnetic focusing

A. I. Berdyugin*, B. Tsim, P. Kumaravadivel, S. G. Xu, A. Ceferino, A. Knothe, R. Krishna Kumar, T. Taniguchi, K. Watanabe, A. K. Geim, I. V. Grigorieva, V. I. Fal'ko*

*Corresponding author. Email: alexey.berdyugin@manchester.ac.uk (A.I.B.); vladimir.falko@manchester.ac.uk (V.I.F.)

Published 17 April 2020, *Sci. Adv.* **6**, eaay7838 (2020)
DOI: 10.1126/sciadv.aay7838

This PDF file includes:

Sections S1 to S6
Figs. S1 to S6
References

Section 1. Determining twist angle.

We used two independent methods to find the actual twist angle θ between the two graphene layers. First, the twist angles were found from $\rho_{\mathrm{xx}}(n)$ and $\rho_{\mathrm{xy}}(n)$ measurements using the position of the secondary neutrality points (NP):

$$\theta = 2\arcsin\left(\sqrt{\frac{\sqrt{3}a^2}{8A}}\right), \qquad \text{(S1)}$$

where a is graphene's lattice constant, n the carrier density, $A = 4/\Delta n$ the superlattice unit cell area, and Δn the position of the secondary NP. For device D1, NP are shown in Figs 4A and 4B in the main text yielding $\Delta n = 8.1 \times 10^{12}$ cm^{-2} and $\theta = 1.87°$. For device D2 it was impossible to reach secondary NPs using electrostatic gating; to find the twist angle in this case we used the fact that NPs can be found by extrapolation of the reciprocal Hall resistivity, $(\rho_{\mathrm{xy}})^{-1}$ as shown in Fig. S1A. The main neutrality point is clearly seen at zero carrier density and two van Hove singularities (vHS) are shown by red arrows. To find the secondary NPs, the linear dependences below and above the vHS were extrapolated as shown in Fig. S1A, with NPs corresponding to the intersections with $(\rho_{\mathrm{xy}})^{-1} = 0$. The positions of the secondary NPs for this device, marked by black arrows, give $\Delta n = 15.7 \times 10^{12}$ cm^{-2} corresponding to θ = 2.60 °.

Another way to determine the twist angle is from the periodicity of Brown-Zak oscillations (*13*, *14*), which are shown in Fig. 4C for D1 and in Fig. S1B for D2. The superlattice area can be found as $A = \Delta\left(\frac{1}{B}\right)\phi_0$, where $\Delta\left(\frac{1}{B}\right)$ is the period of Brown-Zak oscillations in the reciprocal magnetic field, and ϕ_0 the flux quantum. This gives $\Delta\left(\frac{1}{B}\right) = 0.0119 \pm 0.0005$ T^{-1} for D1 and 0.00616 ± 0.00005 T^{-1} for D2 corresponding to θ = 1.87±0.01˚ for device D1 and θ = 2.60±0.01˚ for D2, in good agreement with the values found from Hall resistivity.

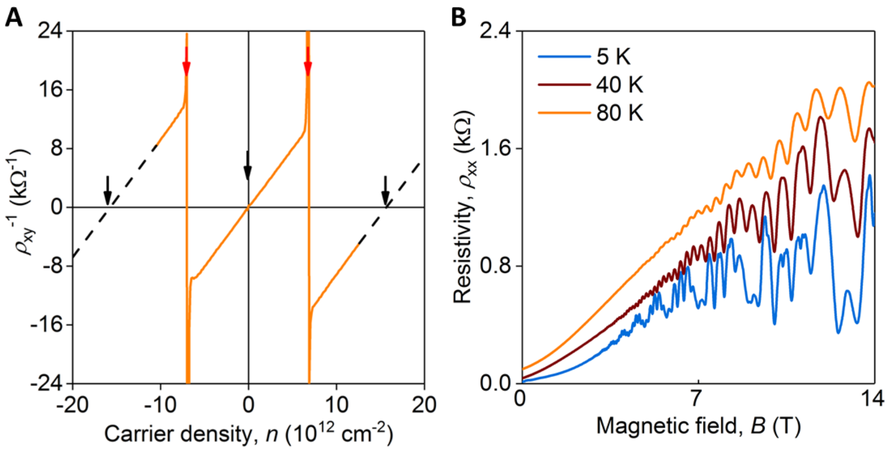

Fig. S1. Hall resistivity and Brown-Zak oscillations in device D2. (**A**), Reciprocal Hall resistivity as a function of carrier density in a magnetic field of 1T measured at 3.5K. Black arrows indicate positions of neutrality points and red arrows show positions of vHS. Black dashed lines are extrapolations that allowed us to find positions of the secondary NPs. (**B**), Longitudinal resistivity vs magnetic field measured at different temperatures for device D2 at the carrier density n = 5.8x10^{12} cm^{-2}.

Section 2. Device fabrication and measurement details

The heterostructures studied in this work were assembled using the standard dry-transfer technique (*24*, *25*), and for the fabrication of the twisted bilayer graphene (TBG) we adapted the tear-and-stack (*26, 4*) method. Details of these methods are outlined below.

First, the top hexagonal boron nitride (hBN) crystal was picked up using a polypropylene carbonate (PPC) polymer spun onto a polydimethylsiloxane (PDMS) film. Then we used a micromanipulator to place the hBN crystal so as to cover only a part of the monolayer graphene located on a SiO_2/Si substrate. Next, hBN was slowly peeled off the substrate, tearing the graphene flake into two pieces while picking up the part covered with hBN. The remaining part of the graphene flake was rotated by 2˚ and picked up with the first half attached to hBN to produce TBG. The temperature of the substrate was kept at 70˚ C throughout this process in order to reduce thermally induced strain or relaxation of the layers. By carefully controlling the micromanipulator, we ensured that graphene layers had no contact with the PPC polymer, guaranteeing a clean interface between the two graphene monolayers. Finally, the bottom hBN crystal was picked up to encapsulate the TBG, and the whole stack released onto a SiO_2/Si substrate. To define 1D contacts, we used reactive ion etching to selectively remove the heterostructures areas, followed by deposition of Cr (3 nm) and Au (60 nm). An additional lithography step was used to make a gold top gate, which also served as an etching mask to define the mesa. An example of the final device is shown in Fig. S2A, where we show one of our dual gated TBG samples. For this sample we used p-doped Si as the bottom gate and Au as the top gate.

Resistance measurements were carried out using the standard low-frequency lock-in technique with a small excitation current ~100 nA; this ensured negligible heating effects down to the lowest measurement temperature, T = 2K. The dual-gated geometry allowed us to control the total carrier density and the displacement field independently. The total carrier density is the sum of carrier densities induced by the top and bottom gates: $n_{\mathrm{total}} = \frac{1}{e}(C_{\mathrm{tg}}V_{\mathrm{tg}} + C_{\mathrm{bg}}V_{\mathrm{bg}})$, where V_{tg} and V_{bg} are the top and bottom gate voltages, e is the electron charge, C_{tg} and C_{bg} are the respective capacitances per unit area of the top and bottom gate (here C_{tg} and C_{bg} were obtained from the Hall measurements). The displacement field is calculated using $D = \frac{1}{2\varepsilon_0}(C_{\mathrm{tg}}V_{\mathrm{tg}} - C_{\mathrm{bg}}V_{\mathrm{bg}})$, where ε_0 is the vacuum permittivity. To achieve a fixed displacement field, V_{tg} and V_{bg} were varied simultaneously according to the above formula, so that only the total carrier density changed. An example of such measurements is shown in Fig. S2B.

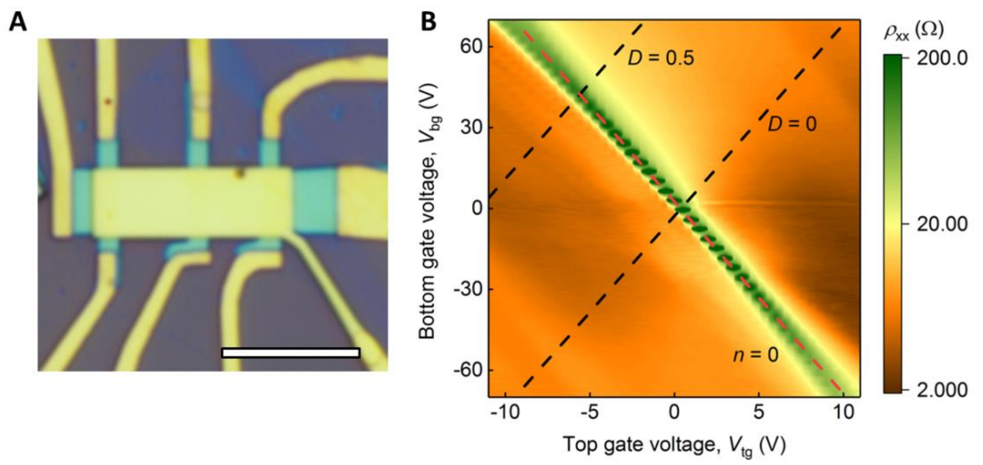

Fig. S2. Dual-gate measurement setup. (**A**), Optical image of device D2. Scale bar, 7 μm. (**B**), ρ_{xx} as a function of top and bottom gate voltages for device D2 at T = 3.5K. Red dashed line corresponds to the conditions of zero carrier density and black dashed lines show the direction of double gate sweeps at D=0 and 0.5 V/nm.

Section 3. Calculations of TBG band structure

To calculate the band structure of TBG, we used the model reported in ref. (*31*), where the Hamiltonian is given by:

$$\hat{\mathrm{H}}_{\mathrm{TBG}} = \begin{pmatrix} \begin{matrix} \frac{\Delta}{2} & \nu\pi^{\dagger} \\ \nu\pi & \frac{\Delta}{2} \end{matrix} & w \sum_{j=0,1,2} e^{-i\,\Delta\mathbf{K}_{\mathrm{j}}\cdot\boldsymbol{r}} \begin{pmatrix} 1 & e^{i\frac{2\pi}{3}j} \\ e^{-i\frac{2\pi}{3}j} & 1 \end{pmatrix} \\ w \sum_{j=0,1,2} e^{i\,\Delta\mathbf{K}_{\mathrm{j}}\cdot\boldsymbol{r}} \begin{pmatrix} 1 & e^{i\frac{2\pi}{3}j} \\ e^{-i\frac{2\pi}{3}j} & 1 \end{pmatrix} & \begin{matrix} -\frac{\Delta}{2} & \nu\pi^{\dagger} \\ \nu\pi & -\frac{\Delta}{2} \end{matrix} \end{pmatrix}. \qquad \text{(S2)}$$

This is equivalent to the continuum-model Hamiltonian derived in ref. (*3*) up to a gauge transformation. The on-diagonal blocks describe the top and bottom layers of graphene where ν is the Dirac velocity for the monolayer and Δ the energy shift induced by the perpendicular displacement field. The off-diagonal blocks describe the interactions between the two layers, with the interlayer coupling strength given by w=110 meV.

The original band touching point of the top layer is placed at the corner of the mini Brillouin zone (mBZ) κ, and the band touching point of the bottom layer is placed at κ'. The vectors $\Delta\mathbf{K}_{\mathrm{j}}$ accounting for the shift between Brillouin zone corners for the two layers (as illustrated in Fig. S3) are given by:

$$\Delta\mathbf{K}_{\mathrm{j}} = \frac{4\pi\theta}{3a}\left(-\sin\left(\frac{2\pi j}{3}\right), \cos\left(\frac{2\pi j}{3}\right)\right), \qquad \text{(S3)}$$

where θ is the twist angle between the two layers in radians and a=2.46 Å is graphene's lattice constant.

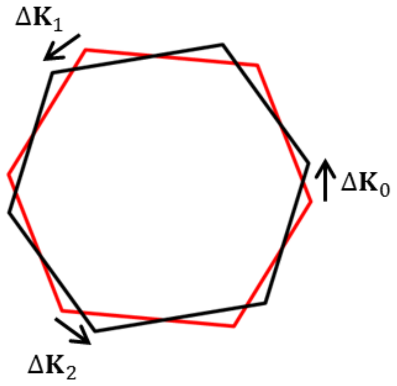

Fig. S3. Brillouin zone of two twisted graphene layers. Red and black hexagons are the original Brillouin zones of the two monolayers overlaid with a relative twist. The vectors $\Delta\mathbf{K}_{\mathrm{j}}$ (defined in Eq. (S3)) account for the shift between the original Brillouin zone corners of the two graphene layers making up a twisted bilayer graphene.

The Hamiltonian in Eq. (S2) is shown for the K valley of the original Brillouin zone. To obtain the band structure in the K' valley, we use a π-rotation of this Hamiltonian.

The miniband spectrum is calculated by zone folding (*32*), i.e., by bringing the states in the K valley with momenta connected by the reciprocal lattice vectors of the moiré pattern to the first mBZ. The basis of *k*-states of the top and bottom layer are formed from the reciprocal lattice vectors $\Delta\mathbf{K}_0 + \mathrm{m}\mathbf{G}_1 + \mathrm{n}\mathbf{G}_2$ and $2\Delta\mathbf{K}_0 + \mathrm{m}\mathbf{G}_1 + \mathrm{n}\mathbf{G}_2$, respectively, where $\mathbf{G}_1 = \Delta\mathbf{K}_0 - \Delta\mathbf{K}_1$ and $\mathbf{G}_2 = \Delta\mathbf{K}_0 - \Delta\mathbf{K}_2$. The number of basis states is chosen to ensure convergence of the first three conduction and valence bands. The resulting Hamiltonian that contains the matrix elements between the basis states is diagonalised using a similar method to ref. (*33*).

Section 4. Numerical simulations of transverse magnetic focusing

To simulate TMF maps shown in Fig. 2 in the main text, we first calculate the band structures to extract the Fermi surfaces and then determine the cyclotron orbits in real space by rotating the Fermi surfaces by 90° and scaling by *ħ/eB (the scaling factor is obtained from the quasi-classical equations of motion (32)). Charge carriers propagate either clockwise or anticlockwise, depending on the sign of the effective charge. We assume specular boundary conditions, so that in a magnetic field the carriers travel along the edge of the sample following cyclotron (skipping) orbits and caustics of skipping orbits focus onto equidistant points. The drift direction of the skipping orbits depends on the effective charge of the carriers and the direction of the magnetic field.*

To achieve consistency with the experiment we select the states that are moving away from the injection point with energies between ε_f *and* ε_f+eV *(where* ε_f *is the Fermi energy and V the applied voltage). The group velocity is calculated from the band structure using* $v \propto \nabla_{\mathbf{k}}\mathbf{E}$*, i.e., it is related to the energy dispersion (for example, the velocity is smaller in flatter parts of the dispersion). Accordingly, as the applied voltage elevates the Fermi level, it results in extra states being occupied such that the available states are populated with a probability proportional to* $|\nabla_{\mathbf{k}}\mathbf{E}|^{-1}$ *and different injection angles should be weighted with a probability proportional to the density of states.*

The TMF spectra shown in Fig. 2B, E in the main text are calculated numerically by using a similar method to ref. (12). This is achieved by counting how many electrons enter contact 3 (Fig. 1C in the main text) having a finite width w. *The non-local resistance* $(\mathrm{V}_3-\mathrm{V}_4)/\mathrm{I}_1$ *is then found by calculating* $(\mathrm{N}_3-\mathrm{N}_4)/\mathrm{N}_1$*, where* N_1 *is the total number of injected electrons,* N_3 *is the number of electrons entering contact 3 and* N_4 *is a smooth background given by* $\mathrm{N}_4=\sum_{\mathrm{i}}^{\mathrm{N}_1}\mathrm{w}/\mathrm{d}_{\mathrm{i}}$. *Here the subscripts correspond to the device contacts in Fig. 1C and* d_{i} *is the distance between consecutive skips along the edge of the* i^{th} *trajectory.*

To investigate whether the TMF spectra are sensitive to the crystallographic orientation of graphene layers with respect to the skipping direction (the edge of the sample), Fig. S4 compares TMF maps simulated for different edge orientations characterised by an angle Φ*. To this end, we* fix the orientation of one of the monolayers so that $\Phi=0°$ corresponds to the zig zag edge and $\Phi=90°$ to the armchair edge. The results for parameters of device D1 at $|n|$ = $6.6\text{x}10^{12}$ cm^{-2} give triangular skipping orbits with the distance between the focusing peaks along the sample boundary weakly dependent on Φ – see Fig. S4. Similar results are obtained for all carrier densities where the Fermi surfaces are anisotropic, i.e., for $|n|$ > $3\text{x}10^{12}$ cm^{-2} where the Fermi surface around the γ point has a pronounced triangular shape (see Fig. 2C in the main text). Corresponding TMF maps show focusing peaks at slightly shifted positions relative to each other. The 3-fold symmetry of the triangular Fermi surface means that the TMF maps should repeat after 60° as is indeed seen for Φ = 30° and 90° in Fig. S4, where the results are identical. At low carrier densities, near the main neutrality point, the Fermi surfaces are almost isotropic and the TMF maps are independent of Φ. The positions of vHSs are independent of Φ as well, in agreement with *ref. (12).*

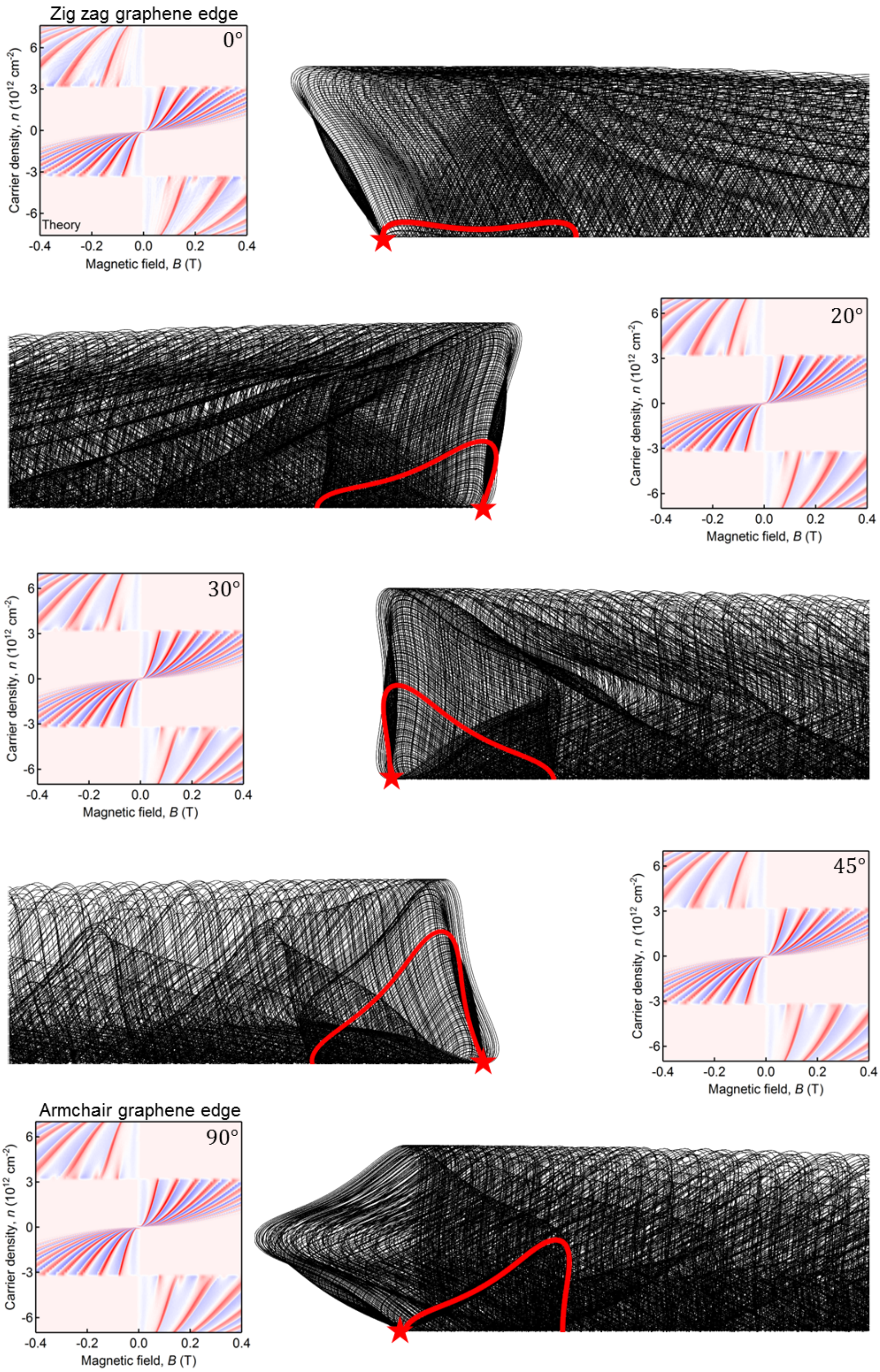

Fig. S4. TMF maps and simulated skipping orbits for different edge alignment. The TMF maps are simulated for $\Phi = \mathbf{0}°, \mathbf{20}°, \mathbf{30}°, \mathbf{45}°$ and $\mathbf{90}°$ for device D1. *T*he orientation of one of the monolayers is fixed such that $\mathbf{0}°$ corresponds to the zig zag edge and $\mathbf{90}°$ to the armchair edge. The skipping orbits are shown at $|n|$ = 6.6x10^{12} cm^{-2}.

Section 5. Electrostatic screening in a finite displacement field

The TMF map in Fig. 2E in the main text shows the effect of a finite displacement field between the two graphene monolayers. To find the effective electric field for each n in this figure, we need to take into account electrostatic screening. At twist angles ~2° and low carrier densities, the two monolayers are almost decoupled. To take into account electrostatic screening in this case, we include a screening term as proposed in ref. (*34, 35*):

$$\frac{ed}{\varepsilon_0 \varepsilon}\left(\varepsilon_0 D - \left(\frac{1+\varepsilon}{4}\right)(n_1 - n_2)e\right) = \frac{hv}{2\sqrt{\pi}}\left(s_1\sqrt{|n_1|} - s_2\sqrt{|n_2|}\right), \qquad \text{(S4)}$$

$$n = n_1 + n_2 \,. \qquad \text{(S5)}$$

where n_1 and n_2 are carrier densities in the two parallel graphene layers separated by a distance d, D the applied displacement field, v the Dirac velocity, the band indices s_1 and s_2 are given by $s_i = n_i/|n_i|$, and the electron charge $e < 0$. In case of the TBG, we use $d \approx 0.34$ nm and following Refs. (*35, 36, 37*), the dielectric constant for twisted bilayer $\varepsilon = 2.7$. The total carrier density n is given by Eq. (S5). To find the effective electric field for each value of n and D used in the experiment, the two equations are solved simultaneously using the Dirac velocity for monolayer graphene, $v=10^6$ m/s. In our calculations, we take n_1 to be the bottom layer and n_2 to be the top layer. The positive direction of D is from the top to the bottom (pointing downwards).

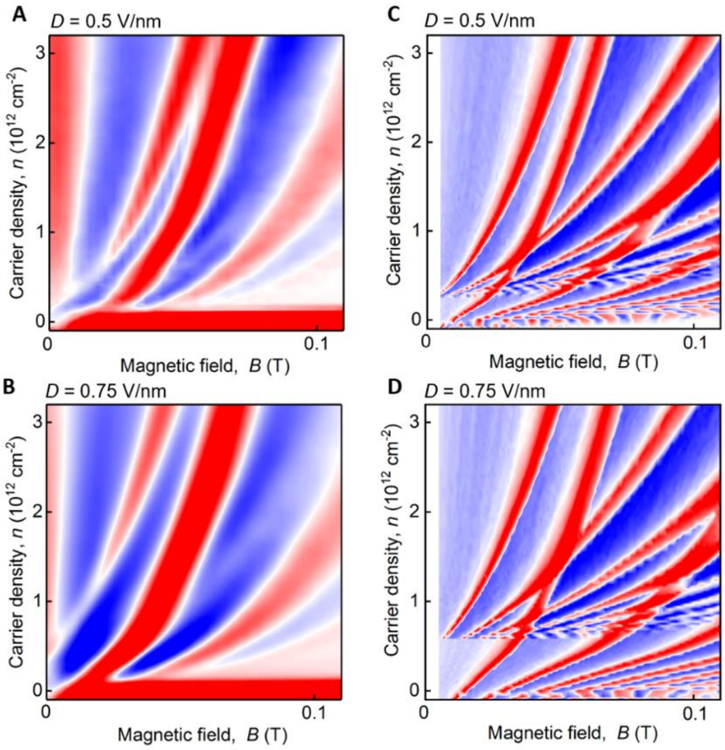

Fig. S5. Further examples of TMF in a displacement field. (**A**), R_f as a function of a magnetic field and carrier density measured for the device D2 at 2K at a distance 4.9 μm from the injector in a displacement field 0.5 V/nm. Colour scale: blue to red ±2.5 Ω. (**B**), R_f as a function of a magnetic field and carrier density measured for the device D2 at 5K at a distance 4.9 μm from the injector in a displacement field 0.75 V/nm. Colour scale: blue to red ±1.5 Ω. (**C**), TMF map calculated numerically for panel (A). (**D**), TMF map calculated numerically for panel (B).

Section 6. Temperature dependence of electron scattering length

Fig. 3B of the main text shows the temperature dependence of the relative scattering length L_S/L_{path} for the electrons near the main and secondary neutrality points, where L_S is the electron scattering length and L_{path} is the length of the trajectories extending from the injector to the first focal point. To extract the absolute scattering lengths we have calculated L_{path} such that the position of the first focal point coincides with the position of the voltage probe. For the electrons near the main neutrality point, $L_{path} \approx \pi \frac{L}{2}$, (see inset in Fig. 1a in the main text), where L is the distance from the current injector to the voltage probe, which is independent of the angle between the crystallographic axes orientation of graphene layers and the sample edge. Near the secondary neutrality point L_{path} is sensitive to the relative orientation of the graphene layers and the sample edge, as can be seen in Fig. S4. We have calculated L_{path} and extracted the corresponding scattering lengths for several characteristic angles between the device edge and zig zag axis of the top graphene layer: $L_{path\,0^\circ} = 1.11\,L$; $L_{path\,10^\circ} = 1.25\,L$; $L_{path\,20^\circ} = 1.58\,L$; $L_{path\,30^\circ} = 1.77\,L$; $L_{path\,45^\circ} = 1.93\,L$. Using these values we extracted the scattering lengths, presuming different crystallographic orientations and compare them in Fig. S6. This showed that in all cases scattering lengths vary between ~100 µm at low *T* and a few µm at *T* =30K, indicating the importance of electron-electron scattering at elevated *T* as discussed in the main text.

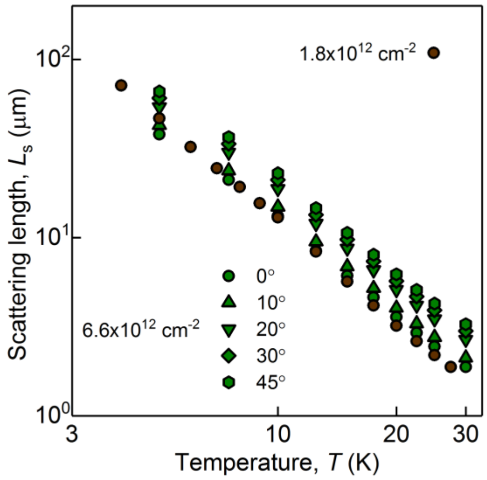

Fig. S6. Temperature dependence of electron scattering length. Electron scattering lengths corresponding to different relative orientations of the graphene's crystallographic axes and the sample edge were extracted from the temperature dependence of the first focusing peak in Fig. 3B of the main text using L_{path} calculated as described in Supplementary section 6.

REFERENCES AND NOTES

1. G. Li, A. Luican, J. M. B. Lopes dos Santos, A. H. Castro Neto, A. Reina, J. Kong, E. Y. Andrei, Observation of Van Hove singularities in twisted graphene layers. *Nat. Phys.* **6**, 109–113 (2010).

2. E. Suárez Morell, J. D. Correa, P. Vargas, M. Pacheco, Z. Barticevic, Flat bands in slightly twisted bilayer graphene: Tight-binding calculations. *Phys. Rev. B* **82**, 121407 (2010).

3. R. Bistritzer, A. H. MacDonald, Moiré bands in twisted double-layer graphene. *Proc. Natl. Acad. Sci. U.S.A.* **108**, 12233–12237 (2011).

4. Y. Cao, J. Y. Luo, V. Fatemi, S. Fang, J. D. Sanchez-Yamagishi, K. Watanabe, T. Taniguchi, E. Kaxiras, P. Jarillo-Herrero, Superlattice-induced insulating states and valley-protected orbits in twisted bilayer graphene. *Phys. Rev. Lett.* **117**, 116804 (2016).

5. K. Kim, A. Da Silva, S. Huang, B. Fallahazad, S. Larentis, T. Taniguchi, K. Watanabe, B. J. Le Roy, A. H. MacDonald, E. Tutuc, Tunable moiré bands and strong correlations in small-twist-angle bilayer graphene. *Proc. Natl. Acad. Sci. U.S.A.* **114**, 3364–3369 (2017).

6. Y. Cao, V. Fatemi, A. Demir, S. Fang, S. L. Tomarken, J. Y. Luo, J. D. Sanchez-Yamagishi, K. Watanabe, T. Taniguchi, E. Kaxiras, R. C. Ashoori, P. Jarillo-Herrero, Correlated insulator behaviour at half-filling in magic-angle graphene superlattices. *Nature* **556**, 80–84 (2018).

7. Y. Cao, V. Fatemi, S. Fang, K. Watanabe, T. Taniguchi, E. Kaxiras, P. Jarillo-Herrero, Unconventional superconductivity in magic-angle graphene superlattices. *Nature* **556**, 43–50 (2018).

8. M. Yankowitz, S. Chen, H. Polshyn, Y. Zhang, K. Watanabe, T. Taniguchi, D. Graf, A. F. Young, C. R. Dean, Tuning superconductivity in twisted bilayer graphene. *Science* **363**, 1059–1064 (2019).

9. M. Yankowitz, J. Xue, D. Cormode, J. D. Sanchez-Yamagishi, K. Watanabe, T. Taniguchi, P. Jarillo-Herrero, P. Jacquod, B. J. Le Roy, Emergence of superlattice Dirac points in graphene on hexagonal boron nitride. *Nat. Phys.* **8**, 382–386 (2012).

10. L. A. Ponomarenko, R. V. Gorbachev, G. L. Yu, D. C. Elias, R. Jalil, A. A. Patel, A. Mishchenko, A. S. Mayorov, C. R. Woods, J. R. Wallbank, M. Mucha-Kruczynski, B. A. Piot, M. Potemski, I. V. Grigorieva, K. S. Novoselov, F. Guinea, V. I. Fal’ko, A. K. Geim, Cloning of Dirac fermions in graphene superlattices. *Nature* **497**, 594–597 (2013).

11. C. R. Dean, L. Wang, P. Maher, C. Forsythe, F. Ghahari, Y. Gao, J. Katoch, M. Ishigami, P. Moon, M. Koshino, T. Taniguchi, K. Watanabe, K. L. Shepard, J. Hone, P. Kim, Hofstadter’s butterfly and the fractal quantum Hall effect in moiré superlattices. *Nature* **497**, 598–602 (2013).

12. M. Lee, J. R. Wallbank, P. Gallagher, K. Watanabe, T. Taniguchi, V. I. Fal’ko, D. Goldhaber-Gordon, Ballistic miniband conduction in a graphene superlattice. *Science* **353**, 1526–1529 (2016).

13. R. Krishna Kumar, X. Chen, G. H. Auton, A. Mishchenko, D. A. Bandurin, S. V. Morozov, Y. Cao, E. Khestanova, M. B. Shalom, A. V. Kretinin, K. S. Novoselov, L. Eaves, I. V. Grigorieva, L. A.

Ponomarenko, V. I. Fal'ko, A. K. Geim, High-temperature quantum oscillations caused by recurring Bloch states in graphene superlattices. *Science* **357**, 181–184 (2017).

14. R. Krishna Kumar, A. Mishchenko, X. Chen, S. Pezzini, G. H. Auton, L. A. Ponomarenko, U. Zeitler, L. Eaves, V. I. Fal'ko, A. K. Geim, High-order fractal states in graphene superlattices. *Proc. Natl. Acad. Sci. U.S.A.* **115**, 5135–5139 (2018).

15. K. L. Seyler, P. Rivera, H. Yu, N. P. Wilson, E. L. Ray, D. G. Mandrus, J. Yan, W. Yao, X. Xu, Signatures of moiré-trapped valley excitons in $MoSe_2/WSe_2$ heterobilayers. *Nature* **567**, 66–70 (2019).

16. K. Tran, G. Moody, F. Wu, X. Lu, J. Choi, K. Kim, A. Rai, D. A. Sanchez, J. Quan, A. Singh, J. Embley, A. Zepeda, M. Campbell, T. Autry, T. Taniguchi, K. Watanabe, N. Lu, S. K. Banerjee, K. L. Silverman, S. Kim, E. Tutuc, L. Yang, A. H. MacDonald, X. Li, Evidence for moiré excitons in van der Waals heterostructures. *Nature* **567**, 71–75 (2019).

17. C. Jin, E. C. Regan, A. Yan, M. Iqbal Bakti Utama, D. Wang, S. Zhao, Y. Qin, S. Yang, Z. Zheng, S. Shi, K. Watanabe, T. Taniguchi, S. Tongay, A. Zettl, F. Wang, Observation of moiré excitons in WSe_2/WS_2 heterostructure superlattices. *Nature* **567**, 76–80 (2019).

18. E. M. Alexeev, D. A. Ruiz-Tijerina, M. Danovich, M. J. Hamer, D. J. Terry, P. K. Nayak, S. Ahn, S. Pak, J. Lee, J. I. Sohn, M. R. Molas, M. Koperski, K. Watanabe, T. Taniguchi, K. S. Novoselov, R. V. Gorbachev, H. S. Shin, V. I. Fal'ko, A. I. Tartakovskii, Resonantly hybridized excitons in moiré superlattices in van der Waals heterostructures. *Nature* **567**, 81–86 (2019).

19. V. S. Tsoi, Focusing of electrons in a metal by a transverse magnetic field. *JETP Lett.* **19**, 70–71 (1974).

20. V. S. Tsoi, Determination of the dimensions of nonextremal Fermi-surface sections by transverse focusing of electrons. *JETP Lett.* **22**, 197–198 (1975).

21. C. W. J. Beenakker, J. G. Williamson, M. E. I. Broekaart, P. H. M. van Loosdrecht, B. J. van Wees, J. E. Mooij, C. T. Foxon, J. J. Harris, Coherent electron focusing with quantum point contacts in a two-dimensional electron gas. *Phys. Rev. B* **39**, 8556–8575 (1989).

22. J. J. Heremans, M. B. Santos, M. Shayegan, Observation of magnetic focusing in two-dimensional hole systems. *Appl. Phys. Lett.* **61**, 1652–1654 (1992).

23. T. Taychatanapat, K. Watanabe, T. Taniguchi, P. Jarillo-Herrero, Electrically tunable transverse magnetic focusing in graphene. *Nat. Phys.* **9**, 225–229 (2013).

24. A. V. Kretinin, Y. Cao, J. S. Tu, G. L. Yu, R. Jalil, K. S. Novoselov, S. J. Haigh, A. Gholinia, A. Mishchenko, M. Lozada, T. Georgiou, C. R. Woods, F. Withers, P. Blake, G. Eda, A. Wirsig, C. Hucho, K. Watanabe, T. Taniguchi, A. K. Geim, R. V. Gorbachev, Electronic properties of graphene encapsulated with different two-dimensional atomic crystals. *Nano Lett.* **14**, 3270–3276 (2014).

25. I. Meric, P. Y. Huang, Q. Gao, Y. Gao, H. Tran, T. Taniguchi, K. Watanabe, L. M. Campos, D. A. Muller, J. Guo, P. Kim, J. Hone, K. L. Shepard, C. R. Dean, One-dimensional electrical contact to a two-dimensional material. *Science* **342**, 614–617 (2013).

26. K. Kim, M. Yankowitz, B. Fallahazad, S. Kang, H. C. Movva, S. Huang, S. Larentis, C. M. Corbet, T. Taniguchi, K. Watanabe, S. K. Banerjee, B. J. Le Roy, E. Tutuc, van der Waals heterostructures with high accuracy rotational alignment. *Nano Lett.* **16**, 1989–1995 (2016).

27. E. H. Hwang, S. Das Sarma, Acoustic phonon scattering limited carrier mobility in two-dimensional extrinsic graphene. *Phys. Rev. B* **77**, 115449 (2008).

28. L. Esaki, L. L. Chang, New transport phenomenon in a semiconductor “superlattice”. *Phys. Rev. Lett.* **33**, 495–498 (1974).

29. K. Leo, P. H. Bolivar, F. Brüggemann, R. Schwedler, K. Köhler, Observation of Bloch oscillations in a semiconductor superlattice. *Solid State Commun.* **84**, 943–946 (1992).

30. C. Waschke, H. G. Roskos, R. Schwedler, K. Leo, H. Kurz, K. Köhler, Coherent submillimeter-wave emission from Bloch oscillations in a semiconductor superlattice. *Phys. Rev. Lett.* **70**, 3319–3322 (1993).

31. P. Rickhaus, J. Wallbank, S. Slizovskiy, R. Pisoni, H. Overweg, Y. Lee, M. Eich, M. H. Liu, K. Watanabe, T. Taniguchi, T. Ihn, K. Ensslin, Transport through a network of topological channels in twisted bilayer graphene. *Nano Lett.* **18**, 6725–6730 (2018).

32. N. W. Ashcroft, N. D. Mermin, *Solid State Physics* (Saunders College, 1976).

33. J. R. Wallbank, A. A. Patel, M. Mucha-Kruczynski, A. K. Geim, V. I. Fal’ko, Generic miniband structure of graphene on a hexagonal substrate. *Phys. Rev. B* **87**, 245408 (2013).

34. H. Schmidt, T. Lüdtke, P. Barthold, E. M. Cann, V. I. Fal’ko, R. J. Haug, Tunable graphene system with two decoupled monolayers. *Appl. Phys. Lett.* **93**, 172108 (2008).

35. S. Slizovskiy, A. Garcia-Ruiz, N. Drummond, V. I. Fal'ko, Dielectric susceptibility of graphene describing its out-of-plane polarizability. arXiv:1912.10067 [cond-mat.mes-hall] (20 December 2019).

36. J. D. Sanchez-Yamagishi, T. Taychatanapat, K. Watanabe, T. Taniguchi, A. Yacoby, P. Jarillo-Herrero, Quantum Hall effect, screening, and layer-polarized insulating states in twisted bilayer graphene. *Phys. Rev. Lett.* **108**, 076601 (2012).

37. B. Fallahazad, Y. Hao, K. Lee, S. Kim, R. S. Ruoff, E. Tutuc, Quantum Hall effect in Bernal stacked and twisted bilayer graphene grown on Cu by chemical vapor deposition. *Phys. Rev. B* **85**, 201408 (2012).
